# Urinary biomarkers of urolithiasis risk in Crohn’s disease: hyperoxaluria, hypocitraturia, and hypomagnesuria in patients with and without ileocolic resection

**DOI:** 10.1093/crocol/otag033

**Published:** 2026-04-22

**Authors:** Vit Navratil, Barbora Pipek, Premysl Falt, Jana Zapletalova, Jan Krivinka, Virginia Solitano, Lumir Kunovsky

**Affiliations:** 2nd Department of Internal Medicine—Gastroenterology and Geriatrics, University Hospital Olomouc, Faculty of Medicine and Dentistry, Palacky University Olomouc, Olomouc, Czech Republic; 2nd Department of Internal Medicine—Gastroenterology and Geriatrics, University Hospital Olomouc, Faculty of Medicine and Dentistry, Palacky University Olomouc, Olomouc, Czech Republic; Digestive Diseases Center, AGEL Ostrava-Vítkovice Hospital, Ostrava, Czech Republic; 2nd Department of Internal Medicine—Gastroenterology and Geriatrics, University Hospital Olomouc, Faculty of Medicine and Dentistry, Palacky University Olomouc, Olomouc, Czech Republic; Department of Medical Biophysics, Faculty of Medicine and Dentistry, Palacky University Olomouc, Olomouc, Czech Republic; 2nd Department of Internal Medicine—Gastroenterology and Geriatrics, University Hospital Olomouc, Faculty of Medicine and Dentistry, Palacky University Olomouc, Olomouc, Czech Republic; Division of Gastroenterology and Gastrointestinal Endoscopy, IRCCS Ospedale San Raffaele, University Vita-Salute San Raffaele, Milan, Italy; Departments of Medicine, Epidemiology and Biostatistics, Western University, London, Canada; 2nd Department of Internal Medicine—Gastroenterology and Geriatrics, University Hospital Olomouc, Faculty of Medicine and Dentistry, Palacky University Olomouc, Olomouc, Czech Republic; Department of Surgery, University Hospital Brno, Faculty of Medicine, Masaryk University, Brno, Czech Republic; Department of Gastroenterology and Digestive Endoscopy, Masaryk Memorial Cancer Institute, Brno, Czech Republic

**Keywords:** IBD, Crohn’s disease, urolithiasis, nephrolithiasis, oxaluria, citraturia, magnesium

## Abstract

**Background/aims:**

Patients with Crohn’s disease (CD) are at an increased risk of urolithiasis, particularly after ileocolic (IC) resection. This study aimed to investigate the prevalence of hyperoxaluria, hypocitraturia, and hypomagnesuria in CD patients with and without IC resection, compared to healthy controls.

**Methods:**

This cross-sectional study included three groups: CD patients who had undergone IC resection (group 1), CD patients with IC involvement but without active inflammation and without resection (group 2), and healthy controls (group 3). Each participant collected a 24-hour urine sample for analysis of oxalate, citrate, and magnesium excretion.

**Results:**

A total of 107 participants were enrolled (34 in group 1, 42 in group 2, and 31 in group 3). The prevalence of hyperoxaluria was 26.5% in group 1, 35.7% in group 2, and 29.0% in group 3, with no significant differences between groups (*P* = .664). Hypocitraturia was more common in CD groups: 35.3% in group 1, 31.0% in group 2, compared to 9.7% in controls, with a significant difference from controls (*P* = .034). A significant difference in hypomagnesuria was found in group 1 compared to groups 2 and 3 (*P* = .001).

**Conclusion:**

While hyperoxaluria prevalence was similar across groups, hypocitraturia and hypomagnesuria were more frequent in CD patients, particularly those with IC resection. Monitoring these biomarkers may help identify at-risk individuals and guide prevention of urolithiasis in CD.

## Graphical abstract

**Graphical Abstract:**
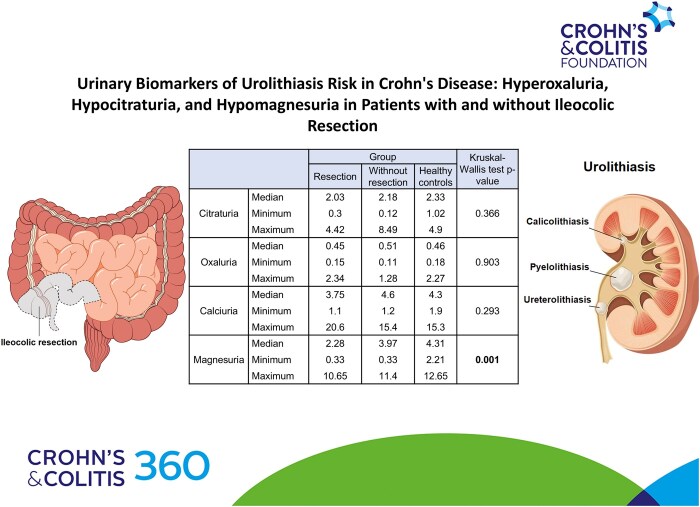

Key Messages **What is already known?** 

- Patients with Crohn’s disease (CD) are at an increased risk of urolithiasis, particularly after ileocolic (IC) resection
- Calcium oxalate stone is the most common type of urolithiasis and increased urinary level of oxalate (hyperoxaluria) is one of its main risk factors
- Urinary excretion of magnesium and citrate may prevent stone formation

**What is new here?** 

- While hyperoxaluria prevalence was similar across groups, hypocitraturia and hypomagnesuria were more frequent in CD patients, particularly those with IC resection.

**How can this study help patient care?** 

- Monitoring these biomarkers may help identify at-risk individuals and guide prevention of urolithiasis in CD.

## Introduction

Crohn’s disease (CD), together with ulcerative colitis (UC), is included in inflammatory bowel disease (IBD).^1^ IBD is commonly complicated by the presence of extraintestinal manifestations (EIM). EIMs shares some pathophysiological mechanisms with IBD due to its immune-mediated mechanism.^2^ Conditions that arise through a different mechanism are referred to as extraintestinal complications (EIC). Urolithiasis, or the formation of stones in the urinary system, is a common EIC of IBD, especially in patients with CD, that could cause significant morbidity and impairment of patients’ quality of life.^3^ Patients with CD have a 2-fold increased risk of urolithiasis compared to the reference population and a 42% higher risk of developing it even before the diagnosis of IBD.^4^^,^^5^ The prevalence of urolithiasis is 12%-28% in CD patients, the risk is especially higher after more extensive intestinal resections.^6^

Ileocolic (IC) resection has a significant impact on the risk of developing urolithiasis. The loss of absorptive surface, especially in the ileum, leads to disruption of the normal absorption of nutrients and electrolytes, resulting in various metabolic changes predisposing one to stone formation.^7^ This results in increased excretion of oxalate in the urine, (hyperoxaluria), one of the main risk factors for the formation of oxalate stones.^8^

Apart from hyperoxaluria, a number of other factors, including dehydration, malabsorption, and changes in urinary pH, are responsible for creating the conditions for urolithiasis.^9^ Furthermore, a reduction in the effectiveness of antilithogenic mechanisms, for example, a reduced level of magnesium (Mg) or citrates in the urine, is important, as they may block the formation and growth of oxalate crystals.^10^^,^^11^ All this, in combination with fluid losses during diarrhea, and a higher concentration of urine, leads to suitable conditions for the formation of urolithiasis.^3^

The aim of this study was to determine the levels of oxaluria, citraturia, and magnesuria in patients with CD, both with and without IC resection, and to compare these levels with those in healthy controls. This study seeks to assess the metabolic changes that predispose CD patients, particularly those who have undergone IC resection, to urolithiasis and to investigate potential biomarkers for identifying patients at higher risk for urinary stone formation.

## Materials and methods

### Study population

We conducted a cross-sectional observational study in one tertiary gastroenterology center focusing on the treatment of IBD. The study was carried out from February 2021 to September 2023. Three groups of individuals were included: Group 1—CD patients with a history of IC resection, group 2—CD patients with IC involvement without a history of bowel resection and without current activity and group 3—healthy volunteers without known inflammatory bowel disease.

Exclusion criteria were age under 19 years, ongoing treatment with citrate, renal failure, defined as grade 4-5 according to the KDIGO classification, that is, glomerular filtration rate <30 mL/min/1.73 m^2^,^12^ history of 3 or more intestinal resections, history of colectomy, short bowel syndrome, current urinary tract infection, and CD relapse, defined as the need for systemic corticosteroid therapy, hospitalization, initiation or change of biological therapy, or other modification of chronic treatment in the past 2 months.

The ethical aspects were considered, the participants signed informed consent, the data were pseudo-anonymized to ensure privacy, and the burden of participating in the study did not exceed the routine procedures the patients usually undergo during the monitoring of their disease. The study was approved by the ethical committee of University Hospital in Olomouc on November 9, 2020.

### Data collection

Anamnesis data were collected using a questionnaire, along with basic anthropometric data and clinical characteristics related to the occurrence of urolithiasis in personal or family history. Additionally, information on current medications, including vitamin C supplementation, and smoking status (assessed semi-quantitatively, including intensity) was gathered. The patients underwent an ultrasound examination of the abdomen with a focus on the kidneys to detect possible lithiasis. At the time of the urine analysis, blood samples were also taken for biochemical examination and urine examination, both from a fresh sample to evaluate urine pH, as well as a 24-hour collection to determine the values of oxalate, citrate, magnesium, and calcium excretion over 24 hours. Before the urine collection, the patients did not follow any special diet and stopped taking magnesium and calcium preparations for a week.

Laboratory examinations were performed using standard methods used in common clinical practice. Absorption spectrophotometry-UV spectrophotometry (ASUR) was used to assess oxaluria, and absorption spectrophotometry with endpoint enzymes at 37 °C (ASEP) was used for citraturia.

### Outcomes and variables definition

The primary outcome was the prevalence of urinary oxalate excretion (oxaluria). Secondary outcomes included the prevalence of urinary citrate, magnesium, and calcium excretion abnormalities (hypocitraturia, hypomagnesuria, and hypercalciuria, respectively) as well as the occurrence of urolithiasis assessed through ultrasound examination.

Hyperoxaluria was defined as urinary oxalate excretion greater than 0.6 mmol/d, based on laboratory reference values of 0–0.44 mmol/d. Hypocitraturia was defined as urinary citrate excretion below 1.6 mmol/d, compared to laboratory reference values of 2.0–5.6 mmol/d.

### Statistical analysis

IBM SPSS Statistics version 24 statistical software (Armonk, NY: IBM Corp.) was used to analyze the data. The chi-square test was used to compare groups in qualitative parameters, or Fisher’s exact test. In quantitative parameters, the groups were compared by the Kruskal–Wallis test. The Mann–Whitney *U* test with Bonferroni correction of significance was used as a post-hoc test. Data normality was tested using the Shapiro–Wilk test. Analysis of key confounders was performed using multivariate logistic regression analysis. All tests were done at the 0.05 significance level.

## Results

A total of 107 patients were enrolled in the study, of whom 34 were CD patients after IC resection (group 1), 42 CD patients with IC involvement without flare and without resection (group 2), and 31 healthy individuals without proven IBD (group 3). The study population consisted of 61 men (57%) and 46 women (43%), with a mean age of 38 years (± 11.5) and a median age of 37 years, with a range of 20 to 74 years. For more detailed characteristics of the population, see Table 1.

**Table 1:** Characteristics of the population.

		Group			Kruskal–Wallis test *P*-value
		Resection	Without resection	Healthy controls	
**Age (years)**	Mean	41.2	34.6	39.6	**.043**
	SD	12.3	10.8	10.7	
	Median	40.6	34.4	37.5	
	Minimum	20.1	19.6	27.2	
	Maximum	74	67.1	66.1	
**BMI**	Mean	25	26.6	25.2	.194
	SD	3.7	4.3	4.3	
	Median	24.1	25.8	24.1	
	Minimum	19.2	19.7	18.4	
	Maximum	33	39.4	34.6	

		Resection		Without resection		Healthy controls		*P*-value
		Number	Percent	Number	Percent	Number	Percent	
**Sex**	Male	12	35.30%	29	69.00%	20	64.50%	**.008**
	Female	22	64.70%	13	31.00%	11	35.50%	
**Obesity. BMI over 30**	No	31	91.20%	33	78.60%	27	87.10%	.338
	Yes	3	8.80%	9	21.40%	4	12.90%	
**Smoking**	No	24	70.60%	32	76.20%	25	80.60%	.579
	≤5 cigarettes	5	14.70%	3	7.10%	4	12.90%	
	>5 cigarettes	5	14.70%	7	16.70%	2	6.50%	
**Urolithiasis in MH**	No	31	91.20%	39	92.90%	30	96.80%	.71
	Yes	3	8.80%	3	7.10%	1	3.20%	
**Urolithiasis in FH**	No	28	82.40%	35	83.30%	28	90.30%	.622
	Yes	6	17.60%	7	16.70%	3	9.70%	
**Vit C**	No	24	70.60%	27	64.30%	23	74.20%	.648
	Yes	10	29.40%	15	35.70%	8	25.80%	

Post-hoc tests	Resection vs no resection	Resection vs healthy controls	No resection vs healthy controls
**Sex**	**0.010**	0.056	1.000

Abbreviations: BMI, body mass index; FA, family history; MH, medical history; SD, standard deviation.

Significantly more women were found in the resection group compared to the non-resection group (64.7% vs 31.0%, *P* = .010); according to post hoc analysis, the difference in gender distribution was significant between groups 1 and 2 (resection and non-resection), otherwise baseline characteristics were comparable.

Patients after IC resection most often had one operation performed, this was the case in 28 patients. Four patients had re-resection after a previous operation, mostly due to a stenosis, and two patients had a right-sided hemicolectomy. The median length of the resected segment of terminal ileum was 20 cm, and the median time from diagnosis to surgery was 76 months. See Table 2.

**Table 2:** Extent and timing of intestinal resections in group 1.

Resection group	Median	Minimum	Maximum
**Length of terminal ileum resection (cm)**	20	6	115
**Time from diagnosis to resection (months)**	76	0	299
**Time from resection to inclusion in the study (months)**	37	3	162

Patients with CD were treated most often with immunosuppressants, followed by biologic therapy and then oral topical corticosteroids; there were no significant differences between the resected and unresected groups (see Table 3). In addition to the patients on monotherapy, there were eight patients on “traditional combination therapy” with a biologic and immunosuppressant, seven with a combination of corticosteroid and immunosuppressant, two with a biologic and corticosteroid, and finally one patient with all three drug groups.

**Table 3:** Anti-inflammatory therapy in enrolled patients with CD.

		Resection		Without resection		Mann–Whitney *U* test *P*-value
		Number	Percent	Number	Percent	
**Oral topical corticosteroids**	No	29	85.3%	33	78.6%	.452
	Yes	5	14.7%	9	21.4%	
**Immunosuppressants**	No	15	44.1%	19	45.2%	.922
	Yes	19	55.9%	23	54.8%	
**Biologics**	No	27	79.4%	30	71.4%	.424
	Yes	7	20.6%	12	28.6%	

Abbreviation: CD, Crohn’s disease.

There was no significant difference between the groups in the level of oxalate excretion in 24 hours and in the level of citrate in 24 hours. Neither was there a statistically significant difference in the prevalence of individuals with significant hyperoxaluria (ie, with excretion above 0.6 mmol/24 h) between groups 1, 2, and 3 (26.5% vs 35.7% vs 29.0%, *P* = .664), while there was already a statistically significant difference in the prevalence of hypocitraturia (excretion below 1.6 mmol/24 h) (35.3% vs 31.0% vs 9.7%, *P* = .042), when compared separately between the groups, the resection group showed a significantly higher prevalence of hypocitraturia compared to the healthy control group (35.3% vs 9.7%, *P* = .043). This was also true when comparing all CD patients collectively with healthy controls (32.9% vs 9.7%, *P* = .013). There is a trend for lower urinary calcium excretion in the group after IC resection, but the difference does not reach statistical significance (*P* = .293). The differences in urinary excretion of magnesium were significant; the results showed a significant difference between the resection group and the non-resection group (median 2.28 vs 3.97 mmol/L, *P* = .047) and especially between the resection group and healthy controls (median 2.28 vs 4.31 mmol/L, *P* = .0003). The non-resection group vs healthy controls did not reach a significant difference. See Table 4.

**Table 4:** Results of urine analysis.

		Group			Kruskal-Wallis test *P*-value
		Resection	Without resection	Healthy controls	
**Citraturia**	Median	2.03	2.18	2.33	.366
	Minimum	0.30	0.12	1.02	
	Maximum	4.42	8.49	4.90	
**Oxaluria**	Median	0.45	0.51	0.46	.903
	Minimum	0.15	0.11	0.18	
	Maximum	2.34	1.28	2.27	
**Calciuria**	Median	3.75	4.6	4.3	.293
	Minimum	1.1	1.2	1.9	
	Maximum	20.6	15.4	15.3	
**Magnesuria**	Median	2.28	3.97	4.31	**.001**
	Minimum	0.33	0.33	2.21	
	Maximum	10.65	11.4	12.65	

Hypocitraturia and hyperoxaluria		Resection		Without resection		Healthy controls		*P*-value
		Number	Percent	Number	Percent	Number	Percent	
**Citraturia**	Over 1.6	22	64.7%	29	69.0%	28	90.3%	**.042**
	Under 1.6	12	35.3%	13	31.0%	3	9.7%	
**Oxaluria**	Up to 0.6	25	73.5%	27	64.3%	22	71.0%	.664
	Over 0.6	9	26.5%	15	35.7%	9	29.0%	

Post-hoc tests	Resection vs no resection	Resection vs healthy controls	No resection vs healthy controls
**Citraturia under 1.6**	1.000	**0.043**	.090
**Magnesuria**	**0.047**	**0.0003**	.455

CD vs healthy controls		CD		Healthy controls		*P*-value
		Number	Percent	Number	Percent	
**Citraturia**	Over 1.6	51	67.1%	28	90.3%	**.013**
	Under 1.6	25	32.9%	3	9.7%	
**Oxaluria**	Up to 0.6	52	68.4%	22	71.0%	.796
	Over 0.6	24	31.6%	9	29.0%	

Oxaluria, citraturia, calciuria, and magnesuria are measured in mmol/24 h.

An univariate analysis of oxaluria in relation to other factors in the study population (gender, age, body mass index, smoking, and medication) was performed. In the group without resection, a positive link between hyperoxaluria and smoking was demonstrated; in the group without resection, a trend towards hyperoxaluria was observed in males, smokers, and those undergoing immunosuppressive treatment, but without reaching statistical significance. Other factors had no clear influence. In multivariate logistic regression analysis for predicting hyperoxaluria with covariates: group (resected, non-resected, control), gender, age, BMI, smoking, and corticosteroid use, none of these parameters is a significant predictor of hyperoxaluria. See Table 5.

**Table 5:** Multivariate analysis for key confounders.

	*P-*value	OR	95% CI for OR	
			Lower	Upper
**Group (control = reference)**	.527			
**Resection vs control**	.567	0.715	0.227	2.257
**No resection vs control**	.597	1.341	0.452	3.975
**Male sex**	.299	0.618	0.249	1.533
**Age**	.935	1.002	0.963	1.042
**BMI**	.878	0.992	0.893	1.101
**Smoking**	.541	1.358	0.509	3.625
**Corticosteroid medication**	.746	1.232	0.350	4.340

Abbreviations: CI, confidence interval; OR, odds ratio.

Ultrasound examination did not detect the presence of urolithiasis in any of the patients. Patients with a medical history of urolithiasis had this issue already resolved. There were seven patients with a history of urolithiasis, three from the resection group, all of them had reduced levels of citraturia and magnesuria, but only one had hyperoxaluria. Three were from group without resection, all had hyperoxaluria and one of them had hypomagnesuria and hypocitraturia. The last one was from the control group and had hyperoxaluria with normal urine Mg and citrate levels.

## Discussion

The formation of urolithiasis is a complex process involving urinary excretion of lithogenic substances, such as calcium and oxalate, in quantities leading to supersaturation. This is followed by crystal nucleation, aggregation and finally stone growth.^13^ The role of oxalate has been studied since the first half of the last century, later the urinary excretion of citrate, magnesium, and calcium was also taken into consideration.^14^^,^^15^ Later, evidence for a relationship between urolithiasis and bowel surgery began to emerge, as numerous papers evaluating urolithiasis and oxaluria in relation to IBD and conditions after resections and bariatric surgery had been published.^16–23^

This cross-sectional monocentric observational study evaluates the prevalence of hyperoxaluria in CD patients after IC resection compared with CD patients with IC involvement without resection. Of the available literature, there are very few studies directly comparing these two subpopulations with CD in the same or similar parameters,^24^^,^^25^ whereas there is an abundance of literature comparing patients with bowel disease (especially IBD, post-resection conditions, and bariatric surgery) with healthy populations.^17–23^

One of the main stimuli leading to the study was the consideration of whether functional knockout of the IC region by intestinal inflammation could lead to the same effect on the risk of urolithiasis as resection of the same region. The involvement in CD is transmural, often leads to stenosis, and is frequently associated with malabsorption. Therefore, the impact of these changes on factors leading to urolithiasis (eg, bile acid and free fatty acid absorption, gut microbiota, and permeability to oxalate) might be expected to be similar to the effect of IC resection.

In the present study, the difference in the prevalence of hyperoxaluria between the groups of patients with CD with and without resection was not significant; paradoxically, it was somewhat higher in patients without resection (mean 0.57 ± 0.37 vs 0.56 ± 0.38 mmol/24 h, *P* = .903), which is contrary to the literature data, where it has been repeatedly shown that oxaluria in patients with CD or IBD in general reaches higher values after IC resection than in patients without resection, with varying degrees of statistical significance.^24–26^

In our study, there was also no significant difference when comparing the two groups of CD patients with healthy controls. The oxaluria in CD patients was 0.57 ± 0.37 mmol/24 h, whereas in controls it was 0.57 ± 0.38 (*P* = .723). This result is also not consistent with data from literature sources, which are showing that patients with CD, or IBD in general, have higher oxaluria values than healthy controls. However, some papers included patients after extensive surgery such as colectomy or other major bowel resections, so comparison of these data with our results could be misleading.^27–29^ Univariate analysis points to a possible relationship between hyperoxaluria and smoking. Lower oxaluria values could therefore also be caused by a smaller number of smokers, especially those smoking more than 5 cigarettes a day, in the studied population (14.7% in the resection group and 16.7% in the non-resection group, compared to 22%-35% in the general population).^30^ Another possible factor is male gender—the predominance of women in the resection group could also influence the result, but this factor did not reach statistical significance here. However, multivariate analysis does not demonstrate the influence of these factors on oxaluria.

Citraturia was assessed in a similar way to oxaluria. The resection group achieved hypocitraturia somewhat more often than the non-resection group, 35.3% vs 31.0%, but the difference was not significant, *P* = 1.000. A statistically significant difference was only achieved when comparing the group without resection, or both groups with CD, with the group of healthy controls, in which hypocitraturia occurred significantly less frequently: 32.9% in patients with CD vs 9.7% in healthy controls, *P* = .013. This result is consistent with some of the literature data, where no significant difference in citraturia between patients with CD with and without a resection.^24^^,^^25^ Some evidence described significantly higher citraturia values in a healthy population compared to CD patients.^28^ Hypocitraturia in CD patients is likely to be due to malabsorption or due to lower urine pH during diarrhoea.

Regarding magnesuria, the resected group had the lowest values, the CD group without resection had the middle values, and the healthy controls had the highest values, 2.86 ± 2.03 mmol/24 h, 3.90 ± 2.18 mmol/24 h, and 5.13 ± 2.83 mmol/24 h, respectively. The difference between the resected group and healthy controls (*P* = .0003) as well as the resected and non-resected groups was significant (*P* = .047), while the non-resected group did not reach a significant difference compared to healthy controls (*P* = .455). This result is in line with expectations as there may be a loss of magnesium through stool after resections. Magnesuria, similar to citraturia, is a protective factor against the development of urolithiasis; thus, their low levels may lead to hypersaturation of urine with oxalate crystals.^31^ Similar results have been reported in other papers, where patients with an intestinal resection, whether due to IBD or other causes, showed significantly lower magnesuria than healthy controls.^24^^,^^28^

Calciuria values were comparable in all three groups (*P* = .293). McConnell arrived at similar results; in his study, the median Ca/creatinine ratio was 0.33 for both CD patients and healthy controls (^28^). In contrast, Parks et al. described significantly lower calciuria in patients with small bowel resection compared to the control population (4.82 ± 0.52 mmol/24 h vs 5.51 ± 0.05 mmol/24 h, *P* < .001), with significantly lower values achieved by patients after colon surgery, especially after colectomy.^24^ Among the study population, only seven individuals had a history of urolithiasis. Of these, five had hyperoxaluria: one following IC resection, three without resection, and one from the control group. Four exhibited both hypocitraturia and hypomagnesuria, including three from the IC resection group and one from the non-resection group. These findings suggest that hypocitraturia and hypomagnesuria may play a more critical role in lithogenesis after IC resection compared to hyperoxaluria. However, the small sample size limits the ability to draw definitive conclusions.

This study’s primary strengths include its cross-sectional design and the comparison of two distinct patient groups: those with and without IC resection. By focusing on CD patients in a real-world setting, it adds to the understanding of the pathophysiology of urolithiasis in this specific population. The inclusion of healthy controls provides further context for interpreting the results and comparing them to unaffected individuals. Moreover, by evaluating a range of urinary markers (oxalate, citrate, magnesium) and considering their interplay, this study offers a comprehensive look at the potential risk factors for kidney stone formation in IBD patients.

The main limitations of our study include its monocentric design and the relatively small sample size. The prevalence of urolithiasis was not significantly different between the groups, which limits how much can be inferred. Patients with active CD were excluded from the study to limit the influence of systemic corticosteroid therapy and overall inflammatory burden; however, it is the absence of this group could explain the discrepancy between our results and older literature data. The patient’s stool culture was not examined, so the possible contribution of dysbiosis could not be taken into account. Patients were not required to follow a specific diet; however, magnesium (Mg) and calcium (Ca) supplementation was temporarily discontinued. Additionally, given that the study population consisted of individuals without symptoms of urolithiasis, we did not include a detailed analysis of dietary Mg and Ca intake. While such dietary factors could potentially explain some of the observed differences between our results and findings in the literature, their influence is likely minimal. For example, Trinchieri’s research suggests that grapefruit juice may offer a protective effect similar to that of potassium citrate.^32^ Despite these limitations, the strengths of our study, such as its rigorous methodology and focus on key biomarkers, provide a solid foundation for understanding the factors influencing urolithiasis in this patient population.

## Conclusion

This study examines urinary oxalate, citrate, and magnesium excretion as key urinary biomarkers in the pathogenesis of urolithiasis in CD. Our findings indicate that the prevalence of hyperoxaluria is similar across CD patients post-IC resection, CD patients with IC involvement but no resection, and healthy controls, suggesting that hyperoxaluria alone may not be the primary factor driving urolithiasis in this population. Notably, according to available data, CD patients are at approximately twice the risk for urolithiasis compared to the general population, which is likely attributable to other underlying factors. Hypocitraturia, though more common in IBD patients than in controls, did not differ significantly between CD subgroups, while hypomagnesuria emerged as a more relevant urinary biomarker for urolithiasis development, especially in resected patients and those with a history of stones.

Based on these results, routine testing of urinary biomarkers such as oxalate, citrate, and magnesium in CD patients post-IC resection may not be necessary for all patients. However, testing could be considered for those with a history of urolithiasis as part of preventive care. Further research with larger patient cohorts is necessary to clarify the role of these urinary biomarkers in predicting urolithiasis risk. These findings highlight the need for personalized clinical approaches, especially for patients with a history of stone formation, in managing and preventing urolithiasis in CD.

## Data Availability

The analysis of the data is included in the manuscript and the tables. Further detailed information is available by the authors upon reasonable request.
